# Chronic Leg Swelling: Uncovering a Rare Hydatid Cyst

**DOI:** 10.7759/cureus.76243

**Published:** 2024-12-23

**Authors:** Somya Goel, Raju K Shinde, Tushar Nagtode, Khushbu Vaidya, Ashish Jivani

**Affiliations:** 1 General Surgery, Jawaharlal Nehru Medical College, Wardha, IND

**Keywords:** cyst hydatid, echinococcus granulosis, helminthic infection, recurrent hydatid cyst, unilateral leg swelling

## Abstract

Hydatidosis is an infection caused by the helminth *Echinococcus granulosus*. The liver and lungs are the most frequently affected organs, primarily due to their roles in filtering blood. Primary hydatidosis of the skeletal muscles is an exceedingly rare condition, often asymptomatic, which can lead to its misdiagnosis as a more common soft tissue tumour. This case report presents a case of primary hydatidosis localized in the vastus medialis muscle of the thigh in a man in his early 70s. The patient exhibited a chronic swelling in his right thigh that persisted for four years, gradually increasing in size and associated with pain. Diagnostic challenges arose due to the atypical site of the cyst, but ultrasound and MRI ultimately confirmed the presence of a hydatid cyst. Thus, it highlights the importance of maintaining a high index of suspicion for hydatidosis, particularly in regions with high endemicity, like India, where early recognition and accurate diagnosis are vital for effective management.

## Introduction

Hydatid cyst, also called hydatidosis, is a global parasitic zoonosis caused by *Echinococcus granulosus* [1]. It is endemic to various countries like North Africa, Turkey, the Mediterranean, Australia, India, South America, Northern China, and the Philippines [2].

India reports an annual incidence of about one to 200 per 100,000 population, which carries a medical and monetary burden on the country [3]. Therefore, the early detection and elimination of the disease becomes a critical priority. The liver and lungs are the most frequently reported as the primary sites of hydatidosis, attributed to their roles as the body's primary and secondary filtration systems [4]. Usually, intramuscular hydatid cysts are rare, ranging from 1% to 5%, and often spread from other areas [5]. Even though most cases do not have clear-cut manifestations of the disease, usually, a painless, slow-growing mass is commonly found as a presenting complaint [2]. However, Jia et al. [6] have documented the first case of primary muscular hydatidosis with a peripheral nerve symptom as an initial manifestation, further showcasing the vagueness of initial symptoms. Nevertheless, *Echinococcus* must be kept in the differentials, especially in endemic areas.

The incidence of hydatidosis presenting as a primary swelling in the musculoskeletal system accounts for only 0.7-3% [7], signifying the rarity of the case. The most commonly resorted investigations are USG and MRI; however, severe cases of primary muscular hydatidosis can be only demonstrated with fine-needle aspiration biopsy [5]. Preoperative suspicion of the disease is crucial to prevent any rupture of the cyst that can impose grave complications. Hence, every case documented is crucial in providing crucial preliminary insight into the disease. Very few cases have been documented in the literature [1,7-10], showcasing the need for an increased index of suspicion to ensure accurate diagnosis, even in regions with high endemicity.

We present a rare case of primary hydatidosis localized in the vastus medialis, where the atypical site posed significant diagnostic challenges.

## Case presentation

A man in his early 70s presented to the outpatient clinic of a tertiary care centre in Central India with chronic swelling in his right thigh, persisting for the past four years. The swelling began insidiously, initially appearing as a small lump approximately 2 x 2 cm in size, and gradually increased to its current size of 8 x 5 cm. The swelling was accompanied by pain, which worsened with movement. The patient also reported a history of close contact with animals and mentioned a previous trauma that occurred in his teenage years.

On examination, the swelling was a solitary, afebrile, non-tender mass of about 8 x 5 cm on the anterolateral aspect of the right thigh. The mass was well demarcated, soft in consistency, with restricted mobility perpendicular to muscle on extension. However, on flexion, the mass becomes non-mobile. The skin over the swelling showed no erythema, and no lymphadenopathy was detected.

Investigation

The patient underwent a complete blood count, which revealed a haemoglobin level of 13.3 g/dL and a normal leukocyte count of 8,600 cells/µL. All other laboratory values were within normal ranges. The chest X-ray performed was normal, with no significant findings on the abdominal ultrasound. Ultrasonography (USG) of the right thigh was performed, revealing well-defined anechoic cystic lesions in the intramuscular plane of the quadriceps femoris. The cyst measured about 33 x 74 x 44 mm, with thin internal septae and two to three daughter cysts adjacent to the mother cyst, which measured about 10 x 10 x 10 mm. The USG raised a suspicion for a hydatid cyst, which was then confirmed on magnetic resonance imaging (MRI).

**Figure 1:**
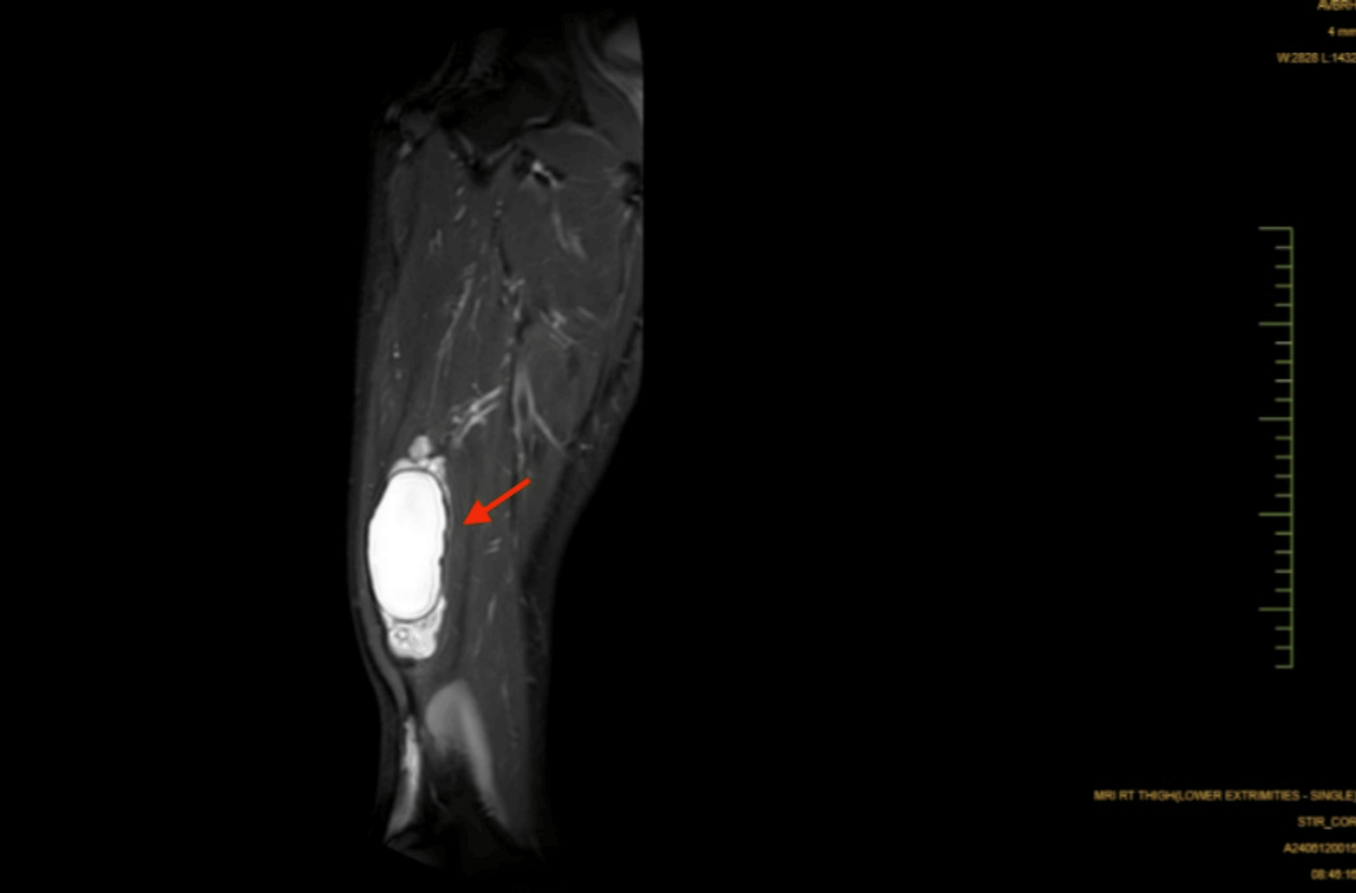
Short-tau inversion recovery (STIR) image of a lobulated, well-defined, oval mass in the quadriceps femoris muscle, specifically in the vastus intermedius

An MRI performed revealed a lobulated, well-defined, round to oval, non-enhancing cystic lesion with altered signal intensity in the quadriceps femoris muscle, specifically in the vastus intermedius. The lesion appeared hypointense on T1-weighted imaging (T1WI) and hyperintense on T2-weighted imaging (T2WI) and short-TI inversion recovery (STIR) sequences (Figure 1).

The largest portion measured approximately 4.9 x 3.7 x 12.2 cm. The lesion also involved the underlying periosteum of the femur with multiple smaller adjacent cysts present surrounding the main lesion. 

Differential diagnosis

The patient came with a history of trauma and a slow-growing mass on his right thigh, which pointed to the possibility of a soft lipoma. However, the association of pain associated with movements ruled out the possibility of a lipoma or the sarcoma of a soft tissue. 

Although rare, a USG of the swelling pointed towards the possibility of a hydatid cyst, which was confirmed by the MRI, which revealed a hypointense lesion on T1-weighted imaging (T1WI) and hyperintense on T2-weighted imaging (T2WI) and short-TI inversion recovery (STIR) sequences, thus confirming the diagnosis of a hydatid cyst.

Treatment

**Figure 2:**
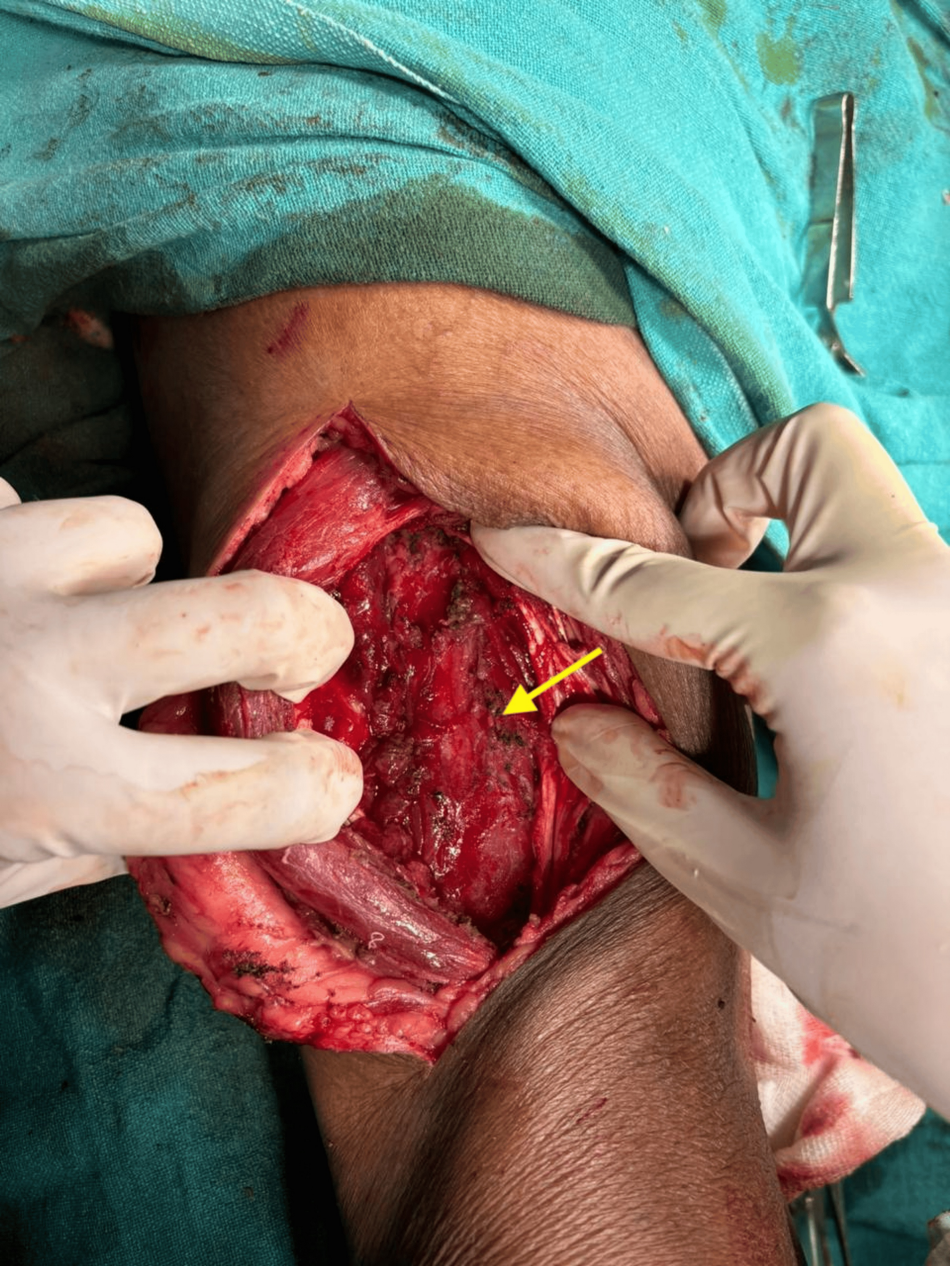
Capsule is visible on retracting the rectus femoris muscle

The cyst was excised under spinal anaesthesia. A lazy-S incision was made over the swelling on the anterolateral aspect of the right thigh. The rectus femoris muscle was laterally retracted, exposing the underlying capsule. The swelling was located beneath the rectus femoris muscle, and a plane was carefully created around it (Figure 2).

**Figure 3:**
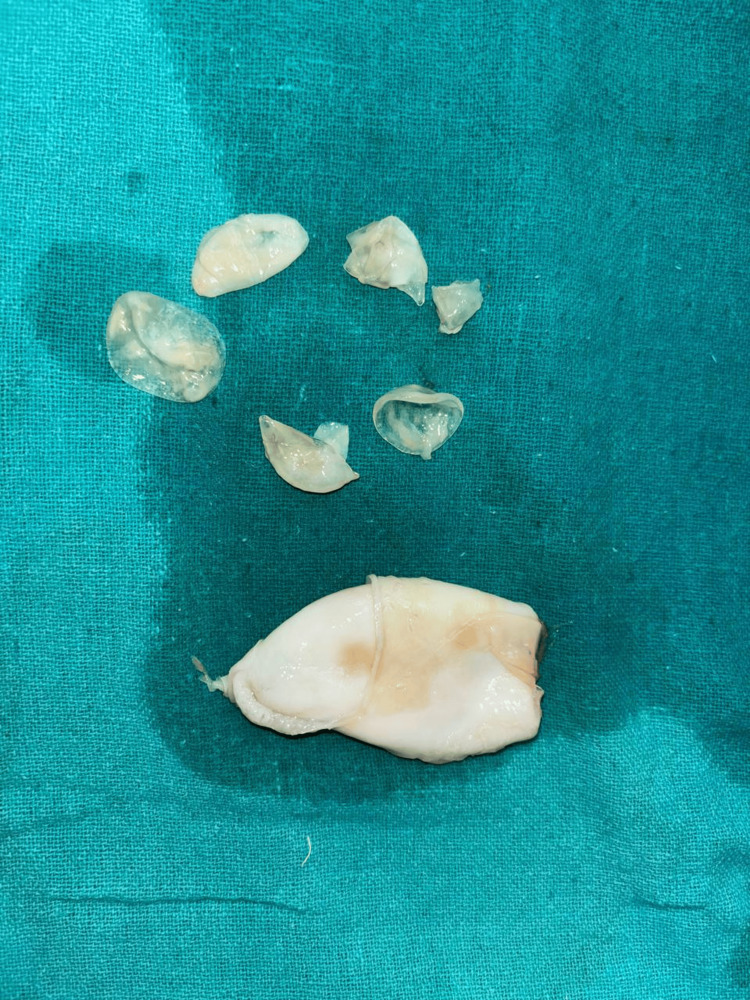
Multiple daughter cysts and hydatid are visible on incising the cyst wall that was excised from the muscle

The pearly white cystic mass, measuring 7 x 5 x 2 cm, was separated from the surrounding adhesions and removed intact and sent for histopathological examination. The histopathology image showed daughter cysts under the microscope (Figure 3).

A linear incision was made on the cyst wall, confirming the presence of a hydatid cyst with a daughter cyst (Figure 3). The wound was thoroughly irrigated with betadine and hydrogen peroxide, and hemostasis was achieved and confirmed. The postoperative course was uneventful, and the patient recovered well. The histopathological diagnosis of the specimen showed features of a hydatid cyst, confirming the diagnosis.

**Figure 4:**
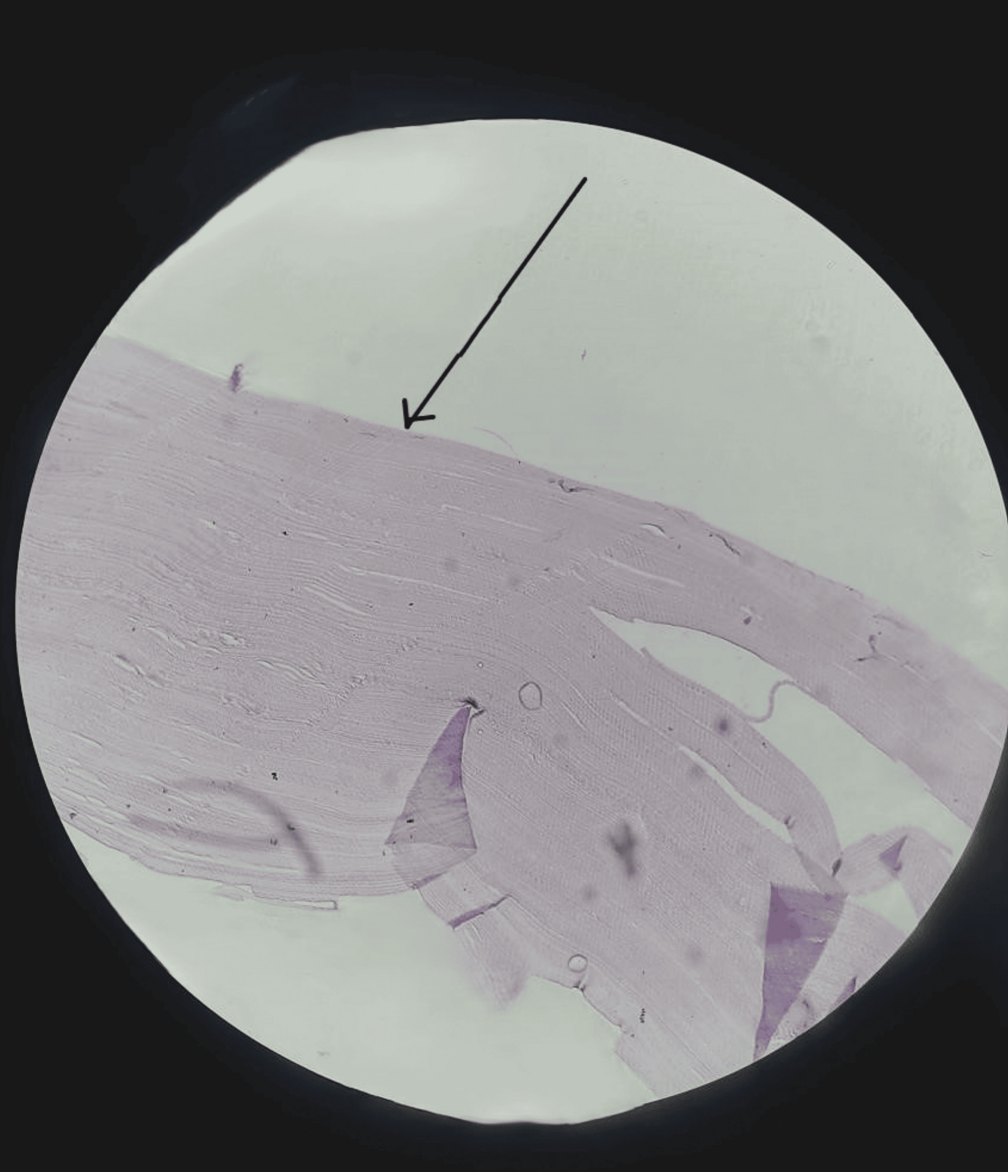
Microscopic image of the capsule shown by the black arrow

Outcome and follow-up 

The patient had no complications in the post-recovery phase and was discharged on day 5 post operation. At one month post follow-up, the patient was healing well with no recurrence.

## Discussion

With a global distribution of two to three million people [11], echinococcosis is a helminthic disease, caused by the larvae of the canine tapeworm *Echinococcus granulosus*. Although humans are incidental hosts, the parasite's life cycle primarily involves dogs as the definitive hosts and sheep as the intermediate hosts. In humans, the liver is the most commonly affected site, accounting for 63% of infections, followed by the lungs, which constitute 25% of cases. Primary hydatidosis occurring in other parts of the body is exceptionally rare, with reports documenting its presence in muscles, bones, kidneys, spleen, and various other locations such as the orbit, breast, thyroid, and urinary bladder [12].

Primary skeletal muscle hydatidosis accounts for less than 1% of all hydatid cysts [1]. This is primarily due to the fact that the lungs and liver serve as primary checkpoints for filtration providing the ideal conditions for the hydatid cyst to grow require oxygen. However, for muscular hydatidosis to occur, the cysts would have to pass through these filter checkpoints. Moreover, the skeletal muscles usually have a higher concentration of lactic acid, which is extremely toxic for the parasite [4], rendering its ability to survive in toxic conditions. In addition, muscle contractility eventually curbs its growth. However, for the one that does localize in the skeletal muscles, it is hypothesized that the volume of the muscle mass and the quality of its blood supply plays a crucial role in determining preferential localization, particularly in the proximal muscles of the lower limbs [13]. It was documented that psoas is the most common muscle for the location of primary hydatidosis [14].

Most often, these cysts are usually present with pain and usually grow by 1 cm every year in the direction of least resistance [9]. A systematic review of 118 patients analyzed the clinical presentations and noted that about 45.9% of all patients presented with pain, while 37 patients presented with pruritis. The swelling was documented in 43 patients, i.e., 39.4 %, and only 3.7% of patients were incidentally diagnosed [5].

Moreover, due to its well-demarcated boundaries, it can be easily confused with soft tissue tumours, like lipomas and sarcoma, as in our case. However, abscesses, myositis, cold abscesses, hematomas, and slow-growing tumours should also be considered in the differentials [14]. Ultrasonography is an excellent screening tool that can be used. However, MRI remains the gold standard for the diagnosis of this pathology [7]. It was demonstrated that USG had a 44.9% specificity, whereas MRI had a significant 77.1% specificity in the diagnosis of the disease with the characteristic 'intensive rim'. With the identification of a primary intramuscular hydatid cyst, total excision is the primary option for treatment [14].

A total of 118 patients have been reported with primary skeletal hydatidosis so far [5]. We add another one of ours to make it to a total of 119.

## Conclusions

In cases of cystic masses within the muscle, especially those presenting without pain but showing localized soft tissue enlargement, the possibility of a primary muscle hydatid cyst should be included in the differential diagnosis. This is particularly important in endemic regions, such as India, especially central India, where hydatidosis is more prevalent. Early recognition and accurate diagnosis in these areas are essential for the effective management and treatment of this uncommon condition. Once confirmed, the most commonly practised treatment is total surgical excision of the cyst, if feasible, to ensure complete removal and prevent recurrence.
